# A Rare Case of Acute Pancreatitis as an Initial Presentation of Acute Myeloid Leukemia

**DOI:** 10.7759/cureus.59108

**Published:** 2024-04-26

**Authors:** Yashwanth M, Arun P, Sanjay S V, Samarth V Shetty, Naveen N C

**Affiliations:** 1 General Medicine, Karnataka Institute of Medical Sciences, Hubballi, IND

**Keywords:** severe anemia, extramedullary manifestation, multiple organ dysfunction syndrome (mods), acute myeloid leukemia (aml), acute pancreatitis

## Abstract

Acute pancreatitis is a rare manifestation of acute myeloid leukemia which can be a presentation at the initial diagnosis or during or after the onset of the disease. Acute myeloid leukemia occurs due to the abnormal proliferation of undifferentiated hematopoietic stem cells in the bone marrow which alter the normal hematopoiesis.

We report the case of a 32-year-old male admitted with a one-month history of fever and backache, followed by 15 days of blackish stool discoloration and two days of abdominal pain and reduced urine output. On clinical examination, he was hypoxic with respiratory distress with epigastric tenderness. Blood investigations and imaging were consistent with acute pancreatitis. A complete blood count with peripheral smear showed severe normocytic normochromic anemia and an increased myeloid series containing 50% myeloblasts and 30% monoblasts. Additionally, some cells displayed cytoplasmic vacuolations, with a reticulocyte count of 2%. These findings were suggestive of acute myeloid leukemia M5. Due to the poor Glasgow Coma Scale (GCS), he was intubated and placed on mechanical ventilation. Unfortunately, he did not improve despite treatment and succumbed to the illness.

## Introduction

Acute pancreatitis occurs due to the activation of pancreatic enzymes, which results in pancreatic inflammation and subsequent destruction of the tissue. The most common symptom of acute pancreatitis is abdominal pain. The most common causes of acute pancreatitis are alcohol abuse, gallstones, drugs, hypercalcemia, and hyperlipidemia [1]. About 20% of patients with an index episode of acute pancreatitis present as acute necrotizing pancreatitis with significant morbidity and mortality [2].

Acute myeloid leukemia is the most common type of acute leukemia in adults. It is due to the abnormal proliferation of undifferentiated hematopoietic stem cells in the bone marrow, which results in damage to the normal blood cells. Most of the time, the clinical presentation is varied, and it is missed, leading to delays in the initiation of treatment resulting in high mortality. The most common type of acute myeloid leukemia is the M2 subtype of the French-American-British classification [3].

## Case presentation

A 32-year-old male presented with a one-month history of fever and backache. He was admitted for similar complaints in a private hospital with a diagnosis of viral fever with thrombocytopenia, where he was transfused with 1 unit of single donor platelets and he was discharged after two days. The patient then developed blackish stool discoloration 15 days ago followed by reduced urine output and abdominal pain for the last two days. Again, he consulted the same hospital, where they again transfused 1 unit of single donor platelets and 2 units of packed red cells. The patient was then later referred to our institute for further management.

As regards the patient's personal and family history, he was a non-smoker and non-alcoholic. There was also no history of similar complaints in the family.

**Figure 1:**
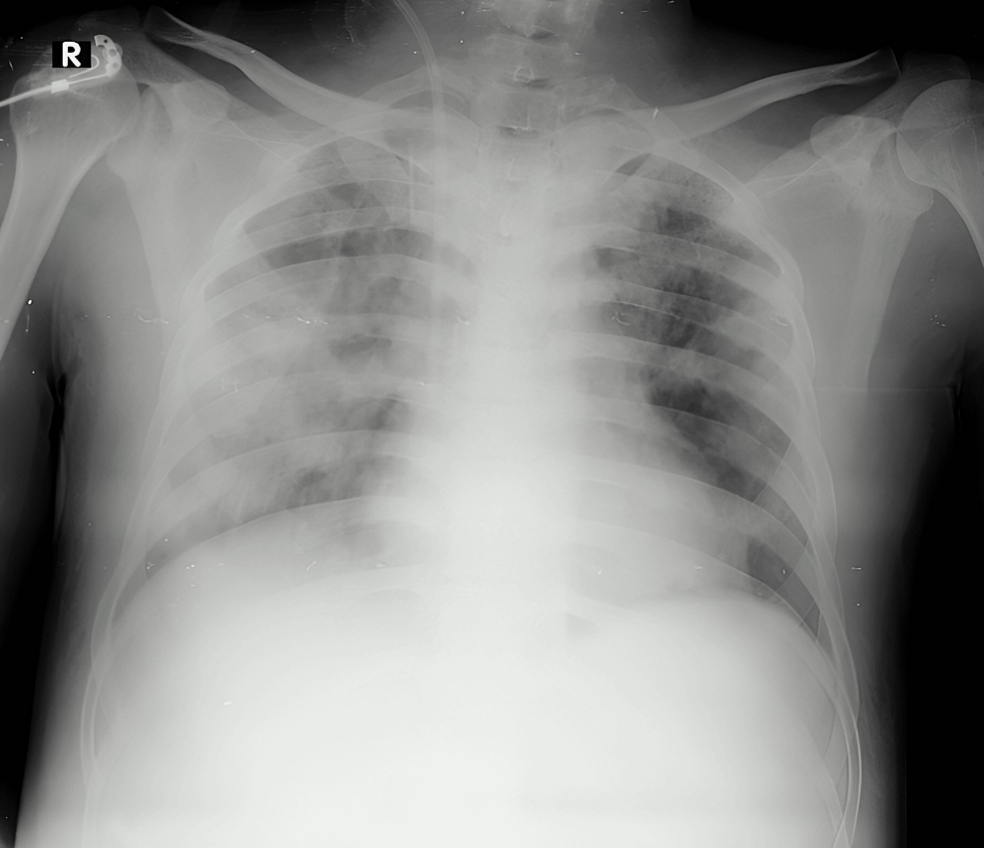
Chest X-ray showing multilobar consolidation

The patient presented to the casualty with a saturation of 82% on room air, a pulse rate of 112 beats per minute, a respiratory rate of 28 cycles/minute, a blood pressure of 130/70 mmHg, and a random blood sugar of 167 mg/dl. On examination, the abdomen was distended with guarding and tenderness in the epigastric region. On the respiratory system, during auscultation, there were bilateral fine crepitations in the infraaxillary and infrascapular areas. He was intubated because of respiratory distress and poor Glasgow Coma Scale (GCS) and put on a mechanical ventilator. Hemodialysis was initiated in view of anuria for 24 hours. A chest X-ray showing multilobar consolidation is shown in Figure 1.

Table 1 depicts the laboratory report showing acute kidney injury, raised uric acid, hyperkalemia, raised lactate dehydrogenase, raised C-reactive protein, hypoalbuminemia, raised aspartate transaminase and alanine transaminase, and very high lipase and amylase. Serology for HIV and HBsAg was negative. Prothrombin time, activated partial thromboplastin time, and international normalized ratio were normal.

**Table 1:** Laboratory report WBC: white blood cell; AST: aspartate transaminase; ALT: alanine transaminase; ALP: alkaline phosphatase; LDH: lactate dehydrogenase; CRP: C-reactive protein

	Day 1	Day 2	Reference values
Hemoglobin (g/dl)	5	5.8	11-15
WBC counts (cells/mm^3^)	29400	31770	4000-11000
Platelet count (cells/mm^3^)	9000	45000	150000-450000
Urea (mg/dl)	59.2	182	10-45
Creatinine (mg/dl)	6.1	8.3	0.5-1.1
Uric acid (mg/dl)	High	High	3.0-7.5
Sodium (meq/dl)	134	130	135-145
Potassium (meq/dl)	5.7	5.8	3.5-4.5
Total protein (g/dl)	4.4	5.1	6-8
Albumin (g/dl)	1.9	2.5	3.5-5
Total bilirubin (mg/dl)	2.9	1.2	0-1.2
Direct bilirubin (mg/dl)	1.9	0.7	0-0.3
AST (U/l)	67	212	Up to 31
ALT (U/l)	58	101	Up to 34
ALP (U/l)	155	370	40-130
Amylase (U/l)		731	Up to 80
Lipase (U/l)		High	Up to 38
LDH (U/l)		High	203-460
CRP (mg/dl)		301.3	0-6
Calcium (mg/dl)		7.8	9-11

**Figure 2:**
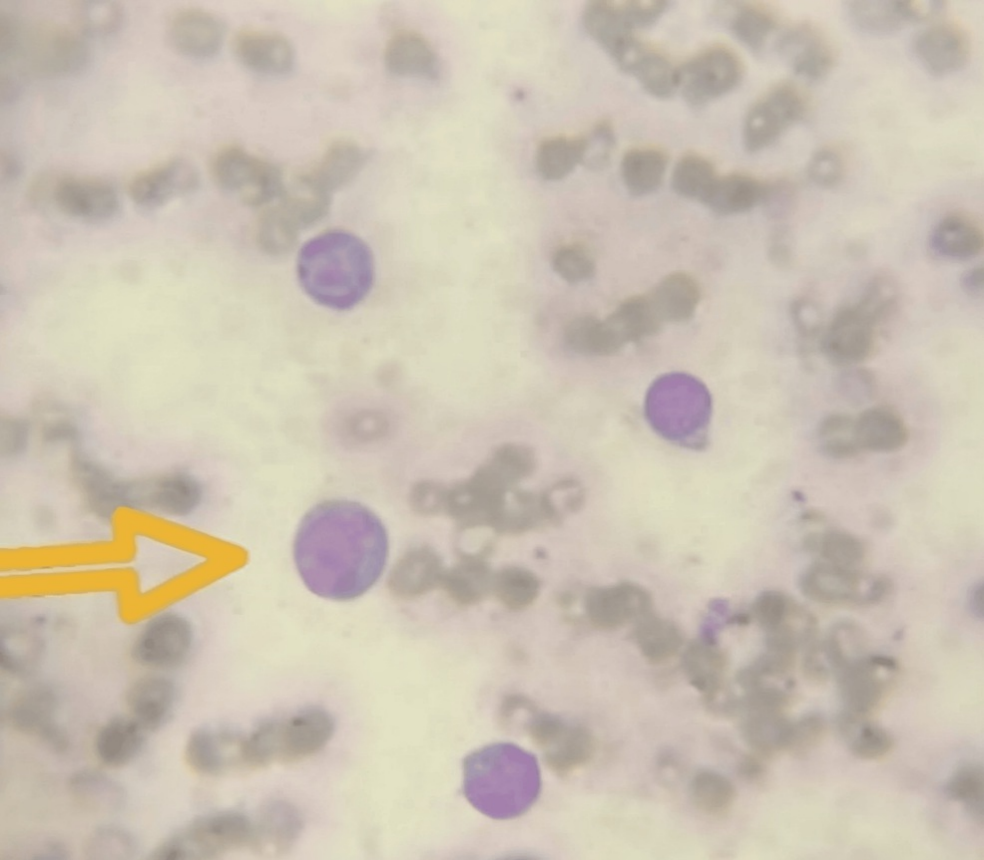
Peripheral smear showing myeloblasts and monoblasts

A complete hemogram with peripheral smear showed severe normocytic normochromic anemia and an increase in myeloid series containing myeloblasts and monoblasts, and some cells show cytoplasmic vacuolations suggestive of acute myeloid leukemia M5 (Figure 2, Table 2).

**Table 2:** Complete hemogram WBC: white blood cell; RBC: red blood cell

Hemogram	Findings	Reference value
WBC counts (cells/mm^3^)	31770	4000-11000
RBC (million cells/mm^3^)	1.96	3.5-5.5
Hemoglobin (g/dl)	5.8	11-15
Platelet count (cells/mm^3^)	45000	150000-450000
Hematocrit (%)	16.2	36-48
Mean corpuscular volume (fL)	82.7	80-98
Mean corpuscular hemoglobin (pg)	29.6	26-32
Neutrophils (cells/mm^3^)	1830	
Lymphocytes (cells/mm^3^)	12180	
Monocytes (cells/mm^3^)	16950	
Eosinophils (cells/mm^3^)	510	
Basophils (cells/mm^3^)	300	
Myeloblasts (%)	50%	
Monoblasts (%)	30%	
Reticulocyte count (%)	2%	

A nephrologist's opinion was taken in view of acute kidney injury secondary to tumor lysis syndrome and advised to continue hemodialysis and allopurinol tablets. A hematologist-oncologist's opinion was taken and advised bone marrow biopsy and cytogenetics, single donor platelet transfusion, and hydroxyurea tablet of 2000 mg per day.

Arterial blood gas analysis shows a high anion gap and severe metabolic acidosis with moderate acute respiratory distress syndrome (Table 3).

**Table 3:** Arterial blood gas analysis pCO2: partial pressure of carbon dioxide; pO2: partial pressure of oxygen

Arterial blood gas analysis	Value	Reference value
pH	7.13	7.35-7.45
pCO2 (mmHg)	25.3	35-45
pO2 (mmHg)	93	83-108
Bicarbonate (mmol/L)	8.4	22-26
Base excess (mmol/L)	19.2	

**Figure 3:**
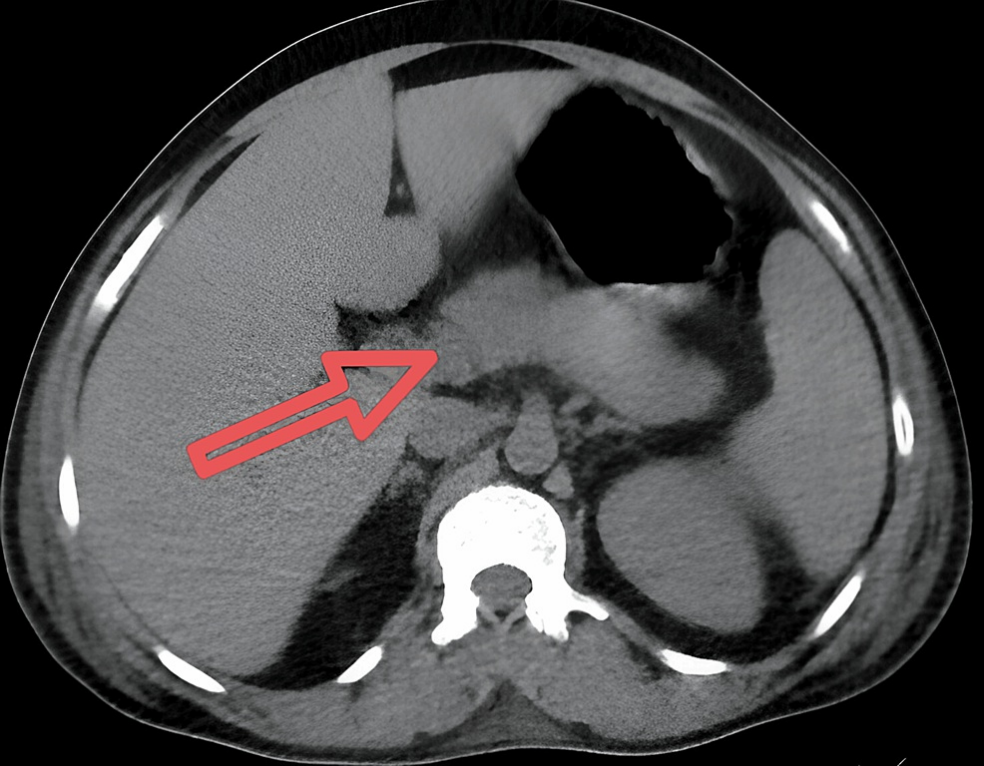
CT of the abdomen showing bulky pancreas

CT KUB (kidneys, ureters, and bladder) was showing multiple scattered fluffy opacities with an air bronchogram, bilateral upper, mid, and lower lobes predominantly involving the upper lobes, minimal pleural effusion on both sides, enlarged liver measuring 22 cm, mild enlargement of the spleen, and bulky-appearing pancreas, measuring 3.1 cm in thickness, suggesting acute pancreatitis (Figure 3).

The patient was treated with broad-spectrum antibiotics, blood products, and inotropes for shock. Unfortunately, he did not improve along the course of treatment and succumbed to death.

## Discussion

Acute pancreatitis associated with acute myeloid leukemia is usually seen when patients are starting chemotherapy using cytarabine or all-trans retinoic acid, which can be managed if chemotherapy drugs are stopped [3]. There are some cases of acute pancreatitis reported after bone marrow transplantation. Few cases reported are due to the direct infiltration of leukemic cells. The extramedullary manifestation of acute myeloid leukemia, which can affect any organ, results in a varied degree of clinical manifestations [1,2].

Acute myeloid leukemia resulting in leukemic cell infiltration into the pancreas is very rare, and the mechanism is still not clearly understood. Some studies have shown that acute myeloid leukemia cells can result in the progression of leukemia by modifying the supportive malignant microenvironment [4].

Acute pancreatitis should always be kept in the differential diagnosis when acute myeloid leukemia patients present with sudden-onset acute persistent pain in the abdomen, regardless of amylase elevation. Early diagnosis and treatment of acute myeloid leukemia can improve the outcome of the patient. Any delay in the diagnosis results in the progression of acute pancreatitis to systemic inflammatory response syndrome and organ dysfunction with rapid progression and very high mortality [3,4].

Yang et al. have reported a similar case of a 61-year-old man with acute myeloid leukemia M2, severe thrombocytopenia, and acute pancreatitis [1].

Although extramedullary infiltration of acute myeloid leukemia is considered a marker of poor prognosis, due to the limited availability of data, it is difficult to determine the prognosis of a patient. Acute pancreatitis as the first extramedullary manifestation is rare, which leads to misdiagnosis [5].

## Conclusions

This case report says that acute pancreatitis is due to acute myeloid leukemia, which is rare but becomes significant in management. Early diagnosis and management can result in better outcomes.
